# Decrease in excitatory neurons, astrocytes and proliferating progenitors in the cerebral cortex of mice lacking exon 3 from the *Fgf2 *gene

**DOI:** 10.1186/1471-2202-9-94

**Published:** 2008-09-30

**Authors:** Kesi Chen, Yasushi Ohkubo, Dana Shin, Thomas Doetschman, L Philip Sanford, Hongqi Li, Flora M Vaccarino

**Affiliations:** 1 Child Study Center, Yale University School of Medicine, 230 South Frontage Rd, New Haven CT 06520, USA; 2 University of Arizona, 1656 E Mabel St, PO Box 245217, Tucson, AZ 85724-5217, USA; 3 Department of Neurobiology, Yale University School of Medicine, 333 Cedar Street, New Haven, CT 06520, USA

## Abstract

**Background:**

The *Fgf2 *gene is expressed in the brain neuroepithelium during embryonic development and in astroglial cells throughout life. Previous knockout studies suggested that FGF2 plays a role in the proliferation of neural progenitors in the embryonic cerebral cortex. These studies exclusively used knockout alleles lacking the *Fgf2 *exon 1. However, the description of putative alternative exons located downstream from the canonical exon 1 raised the possibility that alternatively spliced transcripts may compensate for the lack of the canonical exon 1 in the *Fgf2 *-/- mice.

**Results:**

We generated and characterized a new line of Fgf2 knockout mice lacking the expression of exon 3, which is conserved in all *Fgf2 *transcripts and contains essential heparin and receptor binding interfaces. The expression of *Fgf2 *exon 3 was prevented by inserting a transcriptional STOP cassette in the *Fgf2 *genomic locus. These mice demonstrate a phenotype in the adult neocortex characterized by decreased density and number of cortical excitatory neurons and astrocytes, which is virtually identical to that of the *Fgf2 *-/- mice lacking exon 1. In addition, we also show that the *Fgf2 *exon 3 knockout mice have decreased proliferation of precursors in the adult cerebral cortex, which had not been previously investigated in the other mutant lines.

**Conclusion:**

The results demonstrate that the phenotype of two completely different *Fgf2 *KO mouse lines, lacking exon 1 or exon 3, is remarkably similar. The combined results from these KO models clearly indicate that FGF2 plays a role in cortical cell genesis during embryonic development as well as in adulthood. Thus, FGF2 may be required for the maintenance of the pool of adult cortical progenitor cells.

## Background

FGF2, one of the most potent among the fibroblast growth factor ligands, is expressed in the neuroepithelium of the central nervous system ventricular zone (VZ) from early embryonic development, along with the transmembrane FGF receptors FGFR1-FGFR3. FGF2 promotes growth in the cerebral cortex by increasing the number of cortical progenitor cells in the VZ [1]. The microinjection of FGF2 into the ventricles of E15.5 rat embryos increases cortical volume and the total number as well as density of glutamatergic neurons and astrocytes in the cortex; however, FGF2 treatment does not affect cortical thickness or cortical layer architecture [1]. The *in vivo *injection of FGF2 does not appear to shorten the cell cycle of progenitor cells, indicating that it does not act on the kinetics of cell division. Rather, the increase in total cell number can be attributed to an increase in the number of progenitor cells [1].

The expression of FGF2 is steadily down-regulated during embryonic development [2]. However, FGF2 protein accumulates postnatally in neurons of the CA2 region of the hippocampus and the cytoplasm and nucleus of astroglial cells throughout the brain [3-5]-.

The analysis of *Fgf2 *null (*Fgf2 *-/-) mice demonstrated its unique role in regulating neurogenesis. Specifically, there is a 40% decrease in the number of neurons and astrocytes in the cerebral cortex of *Fgf2 *-/- mice [1], as well as a decreased number of dividing progenitors in the VZ [6]. This supports the earlier finding that FGF2 acts on the progenitor population, and when it is missing, the pool of cortical progenitor cells is significantly decreased. FGF2 may uniquely affect the development of glutamatergic pyramidal neurons in the cortex [7]. Pyramidal neurons identified using SMI-32, latexin, and glutamate antibodies were decreased by 38% in the cortex; in contrast, cortical GABAergic interneurons were not significantly affected. Remarkably, this disruption in excitatory cell number was most significant in the anterior regions of the cerebral cortex [7]. The data are consistent with the notion that FGF2 is important for the development of radial glial cells, which are neuroepithelial progenitors of the VZ that give rise to excitatory neurons and astrocytes during neurogenesis [8]. In addition, FGF2 also seems to control cell migration, since a fraction of the neuronal progenitors remain in the deeper cortical layers and cannot reach layers 2 and 3 in *Fgf2 *-/- mice [9].

*Fgf2 *is encoded by three exons, exon 1, 2, and 3. In the original knockout studies, *Fgf2 *null mice were generated by deleting exon 1 and immediately upstream sequences in the *Fgf2 *gene locus [9-11]-. However, other *Fgf2 *mRNA variants have been described that lack the canonical exon 1 and utilize alternative exons (i.e., exon 1b, 1c) that are located downstream from exon 1 and can be spliced to the second exon [12]. These studies raise the possibility that these alternatively spliced transcripts may compensate for the lack of canonical exon 1 in the *Fgf2 *-/- mice. To answer this question, we generated a new recombinant mouse model that lacks exon 3, an exon that is conserved among the *Fgf2 *transcript variants.

Because exon 3 is over 5 kb long, the current strain of *Fgf2 *knockout mice was generated by inserting a stop codon in front of this exon. This stop sequence was flanked by Cre recognition sites, and as such it is amenable to being excised by Cre recombination, reconstituting the wild type gene. An important question to be addressed is whether these mice would show the same or more profound deficits in CNS development, as compared with the original exon 1 *Fgf2 *knockout line.

## Methods

### Generation and characterization of Fgf2 exon 3 knockout mice

**Figure 1:**
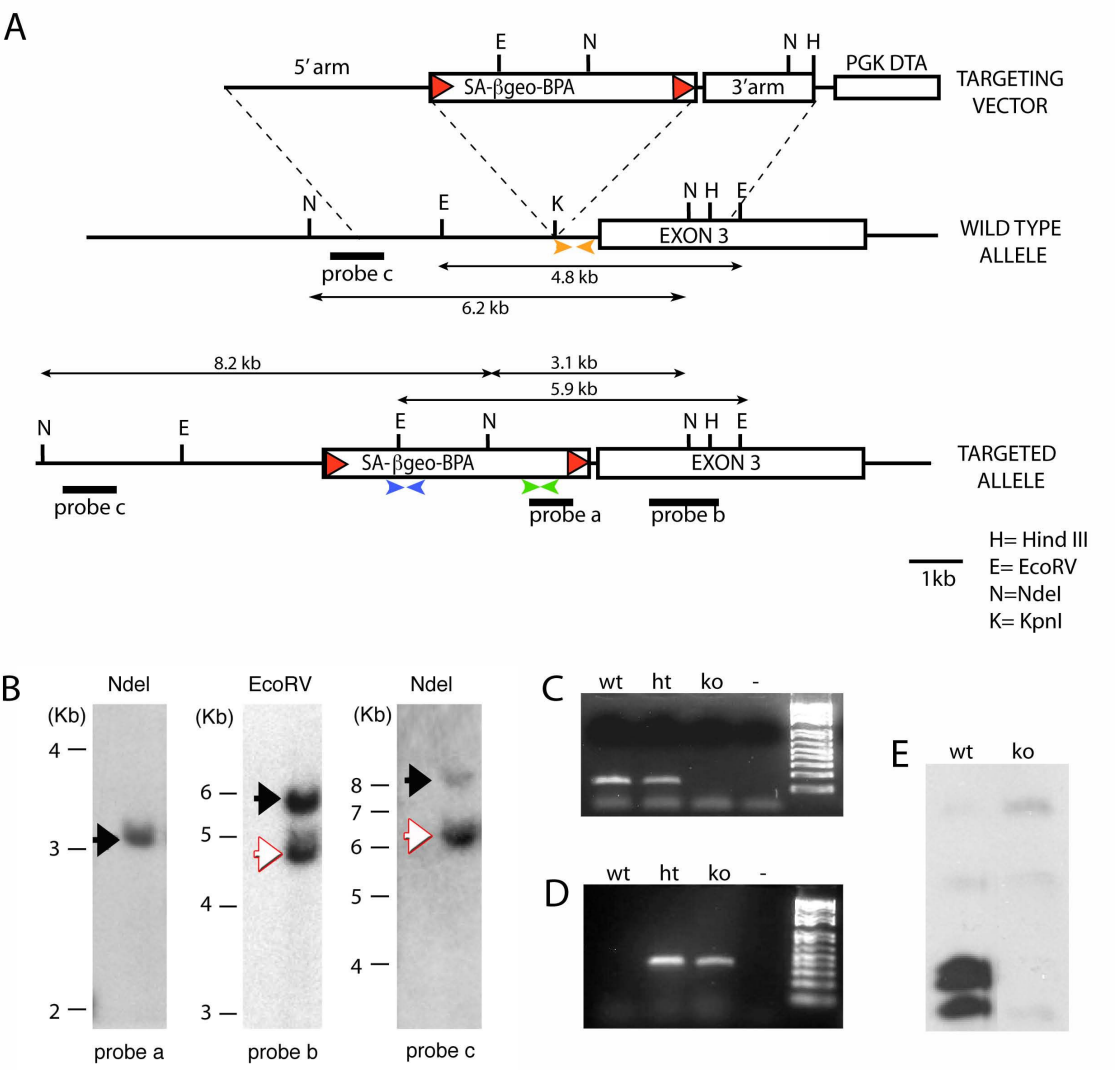
**Generation of *exon3 *knockout by targeted insertion of a transcriptional STOP cassette in the *Fgf2 *locus**. **A**, Structure of the targeting vector, the wild type *Fgf2 *gene and the Fgf2 locus after homologous recombination. Boxes: exons; lines: introns; red triangles: LoxP sites; black bars: location of Southern blot probes; double-arrowed lines: size of the corresponding fragments; orange triangles: location of genotyping primers for the wild type allele; green triangles: genotyping primers for the knockout allele; blue triangles: location of RT-PCR primers to assess the expression of the βgeo product. Abbreviations: SA, Splice acceptor from Adenovirus; βgeo, β-galactosidase -Neomycin gene fusion construct; BPA, polyadenylation site; PGK DTA, Phosphoglycerate kinase promoter-driven diphtheria toxin gene. **B**, Southern blot images of DNA digests from the correctly targeted ES cells. Black arrows point to the targeted allele, and white arrow to the wild type allele. **C**, RT-PCR with primers specific for the wild type allele (upstream primer in exon 2, downstream primer in exon 3). **D**, RT-PCR with primers specific for the knockout allele (located in the βgal construct, blue triangles in A). **E**, Western blot showing absent expression of the FGF2 protein in exon3 KO mice. Wt, wild type mice; ht, heterozygous mice; ko, mice homozygous for the exon 3 truncated allele.

A targeting vector was constructed containing the SAβgeolox2 transcriptional STOP cassette (obtained by Dr. Phillip Soriano, Fred Hutchinson Cancer Research Center, Seattle) [13] flanked by approximately 5 Kb and 3 Kb of upstream and downstream *Fgf2 *genomic sequence, respectively (Fig. 1A). The SAβgeolox2DTA cassette  contained a splice acceptor site from Adenovirus (SA), the entire coding sequence for the β*geo *gene (encoding a protein with both β-galactosidase and neomycin phosphotransferase activity) followed by a polyadenylation site and flanked by loxP sites. This cassette was inserted by homologous recombination into an intronic *KpnI *site, located approximately 1 kb upstream from the third exon of the *Fgf2 *gene (Fig. 1A). After electroporation of the targeting vector into E14TG2a embryonic stem cells (ES cells), correctly targeted ES cells were identified by PCR and confirmed by Southern analysis. Examples of Southern blots used for screening are shown in Fig. 1B. Correctly targeted ES cells were microinjected into C57BL/6 strain blastocysts and chimaeric mice were mated to Black Swiss mice. The *Fgf2 *heterozygous and exon 3 knockout mice were maintained by intercrossing in a Black Swiss background.

To genotype the wild type *Fgf2 *alleles, the following primer pairs were used: upstream primer: 5'-TTGGTACCCTGGAATATTTTAGCCC-3'; downstream primer: 5'-AATAAGTAACCCAGAATATACTGG-3'. These are complementary to sequences in the intron 2 – 3 at the cassette insertion site (see Fig. 1A, orange arrowheads). To genotype the *Fgf2 *exon 3 knockout allele, primer pairs complementary to sequences within the β*geo *gene and polyadenylation site in the insertion cassette were used (upstream primer: 5'-CCTTCTATCGCCTTCTTGACG-3'; downstream primer: 5'-GGTTCCGGATCAGCTTGATTCG-3') (see Fig. 1A, green arrowheads). The following conditions were used: 94°C for 1 min; 58°C for 1 min and 72°C for 1 min and 30 sec for 34 cycles.

To verify the lack of expression of the *Fgf2 *exon 3 in the KO mice, we reverse-transcribed mRNA purified from brain tissue of wild type mice and *Fgf2 *exon 3 KO animals and performed reverse-transcription polymerase chain reaction (RT-PCR) with an upstream primer in exon 2 and a downstream primer in exon 3 (upstream primer: 5'-TACCTTGCTATGAAGGAAGATGG-3'; downstream primer: 5'-TCAGTGCCACATACCAACTGG-3'; fragment size: 139 bp; Fig. 1C). To verify the expression of the STOP cassette, we used primers in the β*geo *gene to amplify a fragment of 346 bp (Fig. 1D). The upstream primer was: 5'-ATCCTCTGCATGGTCAGGTC-3'; the downstream primer: 5'-CGTGGCCTGATTCATTCC 3' (see Fig. 1A, blue arrowheads). RNA isolation was performed using Trizol (Invitrogen, Carlsbad, CA). Brain tissue was obtained from animals after cervical dislocation and tissue was homogenized in Trizol. RNA was precipitated using isopropanol and washed using 75% ethanol. The RNA pellet was then re-suspended in RNase-free water. Reverse transcription was performed using the SuperScript III kit (Invitrogen, Carlsbad, CA). PCR was performed on the resulting cDNA, using the same conditions as those used for genotyping, described above.

### Western blotting

For western blotting, freshly harvested tissue was homogenized in 20 mM Tris, 2 mM EDTA, 2 M NaCl, 1% NP40, PMSF, and FGF2 was extracted according to published methods [14]. A rabbit polyclonal antibody that recognizes FGF2 epitopes encoded in exons 1 and 2 was used (1:1000, Santa Cruz Biotechnology, Santa Cruz, CA). Molecular weights were determined by Precision Plus Protein standards (Bio-Rad Hercules, CA). Visualization was by chemiluminescence (Amersham Bioscience, Piscataway, NJ).

### Immunocytochemistry

Brain samples were obtained by transcardial perfusion with 4% paraformaldehyde (PFA) in 0.1 M Phosphate Buffered Solution (PBS), pH 7.4, followed by post-fixation overnight in 4% PFA and cryprotection in 20% sucrose. Thirty minutes prior to perfusion, the mice were injected with 100 mg/kg 2-bromodeoxururidine (BrdU). Brains were sectioned with a cryostat at 50μm thickness. For each antibody staining, a series of free-floating sagittal sections (1 out every 10) were blocked for one hour in a 0.1 M PBS/10% normal goat serum solution, pH 7.4, containing 0.2% Triton-X-100 and 0.1% Tween 20. Immunohistochemistry was performed with the following antibodies: neuronal-specific nuclear protein (NeuN) (1:500; Chemicon, Temecula, CA), glial fibrillary acidic protein (GFAP) (1:2000, Dako, Carpinteria, CA), T-box brain 1 protein (TBR-1) (1:1000, rabbit polyclonal, Dr. Chris Englund, University of Washington, Seattle, WA) [15], parvalbumin (1:2500, Sigma-Aldrich, MO). For BrdU staining, sections were incubated in 30% formamide in 2× SSC for one hour at 65°C, washed in 2× SSC and incubated for 30 minutes in 2N hydrochloric acid at 37°C. The primary antibody used was BrdU (1:500, Accurate Chemical, Westbury, NY). Sections were then incubated for one hour at room temperature in the appropriate secondary antibody diluted in 5% normal goat serum. The secondary antibodies were the following (all from Molecular Probes, Eugene, OR): Alexa 488-conjugated anti-mouse IgG (1:500); Alexa 594-conjugated anti-rabbit IgG (1:1000); Alexa 488-conjugated anti-rat IgG (1:1000).

### Stereological analyses

Volume and total cell numbers were calculated as described [16] using the optical fractionator method using a computer coupled to a Zeiss Axioskope 2 Mot Plus equipped with a motorized stage, running the StereoInvestigator software (Microbrightfield, Colchester, VT). Double-immunostaining was visualized by a 594 and 488 nm double exposure filter. Nuclear profiles were counted in 3-dimensional counting frames (size, 100 × 100 × 5 μm), using a sampling grid of 500 × 500 μm randomly placed over the drawings of the cerebral cortex. The cortical boundary on each sagittal section was placed to include the frontal cortex (excluding piriform cortex), motor and association cortices, somatosensory and association cortices, and visual cortex (excluding retrosplenial cortex) according to [17]. The upper cortical boundary was at the pia, and the lower boundary was the white matter. Hippocampal regions were not included. Counting frames were placed in the upper portion of the section (1 μm below the surface) to avoid variations due to differential penetration of antibodies. Between 50 and 150 sampling frames were counted in the cerebral cortex per brain. Counts were extrapolated to the entire cerebral cortical volume to obtain total cell number. Total volume was obtained using the Cavalieri method. The experimental conditions were not known to the investigator at the time of quantification to avoid bias in the counting.

## Results

### Generation and characterization of Fgf2 exon 3 KO mice

The SAβgeolox2DTA DNA cassette, containing a splice acceptor, preceding the entire coding sequence for the β*geo *gene, which was followed by a polyadenylation site  was inserted upstream of exon 3 in the *Fgf2 *gene by homologous recombination. In the modified gene locus, β*geo *mRNA is expressed instead of exon 3, after which there is premature polyadenylation of the gene product. The truncated product is thought to be without function, since many of the receptor binding interfaces and most of the heparin binding sites are in exon 3 [18]. With the inclusion of the loxP sites, the *Fgf2 *gene can be reconstituted via Cre recombination, which should excise the SAβgeolox2DTA sequence.

The *Fgf2 *exon 3-deficient mice were born at the normal Mendelian frequency and displayed no alteration of body size, fertility, or survival. To assess *Fgf2 *exon 3 expression, total RNA was extracted from the brain of 5-month-old *Fgf2 *exon 3 KO, wild type and heterozygous mice and reverse-transcribed into cDNA. RT-PCR analyses were carried out using an upstream primer corresponding to sequences in exon 2 and a downstream primer corresponding to sequences in exon 3 of the wild type *Fgf2 *gene. A specific 139-bp amplicon was obtained from *Fgf2 *wild type and heterozygous mice, but not from their KO littermates (Fig. 1C), demonstrating the absence of the exon 3 transcription from the *Fgf2 *exon 3 KO allele. Conversely, the *Fgf2 *exon 3 KO and heterozygous mice expressed the β*geo *gene in the brain, as assessed by RT-PCR, while no such expression was detected in the wild type mice (Fig. 1D). Western blot analysis demonstrated that the *Fgf2 *exon 3 KO mice have virtually undetectable FGF2 protein isoforms as compared to wild type mice (Fig. 1E).

### Phenotypic analyses

**Figure 2:**
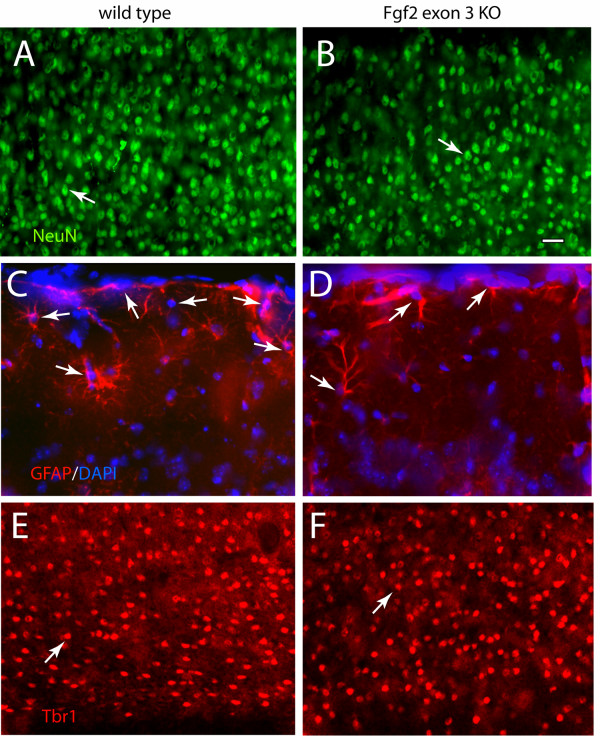
**Immunocytochemical characterization of cellular phenotypes in the cerebral cortex of *Fgf2 *exon 3 knockout mice**. Coronal sections from the parietal regions of the cerebral cortex (above the dorsal hippocampus, approximately 1 mm caudal from the hippocampal comissure) of wild type (A, C, F) and *Fgf2 *exon 3 knockout mice (B, D, F) immunostained for Neuronal Antigen (NeuN) (A, B); Glial Fibrillary Acidic Protein (GFAP) counterstained with DAPI (C, D); and TBR-1 (E, F). A though D are images of layers 1–3 and E, F are images of layers 5–6. Arrows point to nuclei of immunostained cells. Note the decrease in cell density in the knockout. Scale bar, 20 μm.

In order to compare the phenotype of *Fgf2 *exon 3 KO mice with that of *Fgf2 *exon 1 knockout mice, we carried out a detailed analysis of the cerebral cortex in young adult littermate mice (age range, from 2.0 to 5.0 months) homozygous for the wild type or the *Fgf2 *exon 3 KO allele. Cortical neurons and astroglial cells, identified by NeuN and GFAP immunostaining, respectively (Fig. 2), were quantified by stereological analyses. Cortical neurons were decreased by 23% and neuronal density was decreased by 37% in the *Fgf2 *exon 3 KO mice (Fig. 2A, B; Table 1). In addition, astroglial cells were decreased by 34%, and astroglial cell density was decreased by 42% (Fig. 2C, D; Table 1). In contrast, cortical volume was not significantly changed (wild-type volume, 3.2 ± 0.5 × 10^10 ^μm^3^, knockout volume 3.0 ± 1.5 × 10^10 ^μm^3^, p > 0.5). To assess the number of actively proliferating cells, BrdU was administered 30 min before perfusion and the incorporation of the nucleotide in S-phase cells, visualized by immunostaining, was quantified by stereology in the whole cerebral cortex. Proliferating cells were decreased by 29% in the *Fgf2 *exon 3 KO mice as compared to their wild type littermates (Table 2).

**Table 1:** *Fgf2 *exon 3 -/- mice show a decrease in cortical neuron and glial cell populations

	**Cell number**		
	
	**Wild type**	***Fgf2 -/-***	**% of control**
Total NeuN+ cells (10^6^)	1.7 ± 0.04	1.3 ± 0.09*	77
Total GFAP+ cells (10^6^)	1.0 ± 0.08	0.7 ± 0.06*	66
Density NeuN+ cells (10^3 ^/mm^3^)	53 ± 4.8	33 ± 3.3**	63
Density GFAP+ cells (10^3 ^/mm^3^)	33 ± 3.6	19 ± 0.33*	58

Cell number was assessed by stereological analyses. N = 4 per group,*p < 0.05, **p < 0.01 (Student's t-test).

**Table 2:** *Fgf2 *exon 3 -/- mice show a decrease in proliferative cell populations

	**Cell Number**		
	
	**Wild type**	***Fgf2 -/-***	**% of control**
Total BrdU+ cell (10^3^)	5.5 ± 0.40	3.9 ± 0.32*	71
Density BrdU+ cells (10^3 ^/mm^3^)	2.3 ± 0.33	1.4 ± 0.090	60

Cell number was assessed by stereological analyses. N = 4 per group, *p < 0.05 (Student's t-test).

We next ascertained the role of FGF2 in regulating the production of particular subgroups of neurons. In the previous study examining *Fgf2 *exon 1 KO mice, it was shown that the lack of FGF2 caused a 38% decrease in number of glutamatergic cortical pyramidal neurons as well as in the size of their cell bodies [7]. In the current study, antibodies to the transcription factor TBR-1 [15] were used to identify glutamatergic neurons in the cortex (Fig. 2E, F). Stereological analyses revealed a 19% decrease, which is less striking than the data from the previous study, but statistically significant (Table 3). In contrast, the number of parvalbumin (PV)-expressing GABAergic interneurons was not significantly affected (Table 3). Based on qualitative analysis, the decrease in pyramidal cells seemed uniform between all cortical layers and areas of the cortex.

**Table 3:** Neuronal subtypes in the cerebral cortex of *Fgf2 *exon3 null mice

	**Cell Number**		
	
	**control**	***Fgf2 -/-***	**% of control**
Total TBR-1+ cells (10^6^)	1.7 ± 0.42	1.4 ± 1.1*	81
Density TBR-1+ cells (10^3 ^/mm^3^)	55 ± 4.6	4.7 ± 2.4	85
Total PV+ cells (10^6^)	0.84 ± 0.11	0.65 ± 0.05	78
Density PV+ cells (10^3 ^/mm^3^)	26 ± 3.8	22 ± 1.6	82

Cell number was assessed by stereological analyses. N = 4 per group,*p < 0.05 (Student's t-test).

## Discussion

We produced mice with premature termination of the conserved *Fgf2 *exon 3 due to insertion of a transcriptional STOP cassette. Exon 3 contains receptor-binding and heparin-binding domains, therefore its absence is likely to result in loss of function. Furthermore, the β*-gal-neomycin *gene product is expressed in place of exon 3 in these mice, resulting in loss of FGF2 immunoreactivity, likely due to a major conformational change of the protein. Alternatively, the aberrant FGF2-βGEO fusion protein may be unstable and subjected to degradation. The faint band at ~18 kD in the exon 3 lane (2% of the intensity of the 18 kD band from the wild type mouse) is likely non-specific because were it to have resulted from read-through of the truncating stop codon, it would also appear at the 21 kD and 22 kD positions since all three isoforms are translated from the same transcripts, and it does not (0% of the intensity of the wildtype 21 kD and 22 kD bands).

This study shows that the lack of *Fgf2 *exon 3 causes significant deficits in several major cortical cell populations. Overall, cortical NeuN+ neuron number was decreased by 23% and that of GFAP+ astrocytes by 34%; one subpopulation of cortical GABAergic interneurons, the PV+ interneurons, was not significantly affected, although we cannot exclude that a larger sample may reveal a deficit in this population, as well. This replicates the results obtained in our previous study of *Fgf2 *null mice [7], which were generated by deleting exon 1 in the *Fgf2 *gene locus. In that study, NeuN+ cells were decreased by 24% and glial cells by 38%. Thus, the degree of deficit in these two cell populations in the two *Fgf2 *KO models is strikingly similar. The decrease in GFAP+ cells in the adult cortex is also in agreement with the results previously reported by another group [19]. Also, in agreement with the present results, all prior studies of *Fgf2 *KO mice reported a minimal decrease or no significant change in total cortical volume. Given the decrease in neuron number, it is conceivable that some compensatory mechanism maintains normal cortical volume; an expansion of dendritic or synaptic neuropil is a possibility.

Another group reported findings on the cortical phenotype of *Fgf2 *KO mice that are slightly different from those described above [9]. Dono et al. reported a 25% reduction in the number of PV+ neurons, but no change in GFAP+ astroglial cells in the cortex of their *Fgf2 *KO mice lacking exon 1. Possible reasons for this discrepancy include the genetic background (C57BL/6J/129/Sv versus Black Swiss/129/Sv in our mice) and the quantitative methods used for estimating cell numbers. We examined both total number and density of cells by stereology using the optical fractionator and unbiased sampling methods, whereas Dono et al. assessed cell density, presumably by counting cells in selected sections.

The decrease in excitatory neurons expressing TBR-1 is less than what was previously found in the exon 1 KO mice using immunostaining for the neurotransmitter glutamate. However, TBR-1 + excitatory neurons represent only a subset of pyramidal cells, those predominantly found in layer 6 in adult mice. The data suggest that FGF2 may be relatively more important for the genesis of pyramidal cells in other cortical layers.

In our prior exon1 *Fgf2 *KO study [7] the mutants showed no significant decrease in cortical interneurons, as assessed by GABA immunostaining. In the current study, we also found no significant decrease in GABAergic neuron types expressing PV in the cortex. Although the data tend to confirm the lack of a phenotype among the GABAergic cell population, we cannot exclude that a non-PV+ subtype of GABAergic cells is affected by the lack of FGF2. Recent studies from our laboratory suggest that the conditional disruption of FGFR1 resulted in a clear loss of PV+ and somatostatin+ interneurons in the cerebral cortex [20]. This disparity suggests that other FGF ligands may be important for the development of cortical GABAergic cells.

The implications of these results are manifold. First, the results demonstrate that the phenotype of two completely different *Fgf2 *KO mouse lines, lacking exon 1 or exon 3, is remarkably similar. The combined results from these KO models clearly indicate that FGF2 plays a role in cortical cell genesis in embryos as well as in adulthood. This is shown by the similar phenotype in the two lines: a decrease in total cortical neuron number, due to a deficit in pyramidal cells as well as in astrocytes, as shown by NeuN, TBR-1 and GFAP immunostaining. Pyramidal neurons and astrocytes are progeny of radial glial cells, which are located in the VZ [8]. This is consistent with the previously demonstrated loss of proliferative progenitors in the VZ during embryonic development in *Fgf2 *exon 1 KO mice [6]. Another cell type that is drastically curtailed in the *Fgf2 *KO mice are proliferating stem/progenitor cells in the adult subventricular zone [21], which are, too, progeny of radial glial cell [22]. It can be hypothesized that this loss in radial proliferative "units" explains the decrease in number of excitatory pyramidal neurons, astrocytes and SVZ neural stem cells.

The current results also show that FGF2 is required for normal cell proliferation in the mature cortex. In previous studies on exon 1 KO mice, proliferating cells were assessed only in embryos [1,6]. The current study indicates for the first time that FGF2 is required for the maintenance of the pool of progenitor cells that populate the cortical parenchyma in adulthood.

## Conclusion

This study illustrates a novel way of preventing the expression of a specific exon of the *Fgf2 *gene by inserting a transcriptional STOP cassette by homologous recombination in the *Fgf2 *genomic locus. Previous research has shown that there are three putative splice variants of exon 1: 1a, 1b, and 1c [12]. Exon 1a, the canonical exon, was deleted in the previous *Fgf2 *KO models [9-11]-. Alternative splicing of exon 1, in principle, could have given rise to different products exerting compensatory roles in the absence of exon 1a in the previously described *Fgf2 *KO mice. However, whether these FGF2 variants are actually transcribed into mRNA and protein is unclear. The generation of an exon 3 KO in the current study sought to verify the role of FGF2 in development because exon 3 is conserved in the all known FGF2 variants. The results clearly show that the phenotype of the *Fgf2 *exon 3 KO mice show no greater severity, and that the decrease in cell populations is very similar to that found in the exon 1 studies. Overall, the role of FGF2 in regulating cortical development is confirmed by removing the conserved exon 3. The relatively mild phenotype in this *Fgf2 *mutant may be explained by compensatory or redundant relationship with other FGF family genes. Furthermore, this line will allow for future studies in which the intact *Fgf2 *gene can be reconstituted by Cre recombination at selective stages in development or adulthood.

## Authors' contributions

KC carried out the characterization of the brain phenotype of *Fgf2 *exon 3 KO mice by molecular, immunohistochemical and stereological analyses and drafted the manuscript. YO participated in the molecular analyses for the characterization of the phenotype of *Fgf2 *exon 3 KO mice. DS generated the targeting vector for the *Fgf2 *exon 3 KO mice and carried out the screening of the targeted ES cells. TD participated in study design, helped with data analysis and helped to write the manuscript. LPS generated the knockout mice in the University of Cincinnati Gene-Targeted Mouse Service. HL carried out the Western blot assays. FMV conceived the study, participated in its design, helped with data analyses and interpretation and helped to write the manuscript.

All authors read and approved the final manuscript.
